# Current Progress and Future Directions for Theory and Research on Savoring

**DOI:** 10.3389/fpsyg.2021.771698

**Published:** 2021-12-14

**Authors:** Fred B. Bryant

**Affiliations:** Department of Psychology, Loyola University Chicago, Chicago, IL, United States

**Keywords:** savoring, positive emotion regulation, happiness, well-being, positive psychology

## Abstract

As research on savoring has increased dramatically since publication of the book *Savoring: A New Model of Positive Experience* (Bryant and Veroff, 2007), savoring has gradually become a core concept in positive psychology. I begin by reviewing the evolution of this concept, the development of instruments for assessing savoring ability and savoring strategies, and the wide range of applications of savoring in the psychosocial and health sciences. I then consider important directions for future theory and research. To advance our understanding of how naturalistic savoring unfolds over time, future work should integrate the perceptual judgments involved in not only the later stages of attending to and regulating positive experience (where past research has concentrated), but also the initial stages of searching for and noticing positive stimuli. Whereas most research has investigated *reactive* savoring, which occurs spontaneously in response to positive events or feelings, future work is also needed on *proactive* savoring, which begins with the deliberate act of seeking out or creating positive stimuli. To advance the measurement of savoring-related constructs, I recommend future work move beyond retrospective self-report methods toward the assessment of savoring as it occurs in real-time. The development of new methods of measuring meta-awareness and the regulation of attentional focus are crucial to advancing our understanding of savoring processes. I review recent research on the neurobiological correlates of savoring and suggest future directions in which to expand such work. I highlight the need for research aimed at unraveling the developmental processes through which savoring skills and deficits evolve and the role that savoring impairments play in the etiology and maintenance of psychopathology. Research is also needed to learn more about what enhances savoring, and to disentangle how people regulate the intensity versus duration of positive emotions. Finally, I encourage future researchers to integrate the study of anticipation, savoring the moment, and reminiscence within individuals across time.

*No day comes twice. Each moment savored more precious than a span of jade.*

—Zen tradition

## Overview

Savoring has been defined as “the capacity to attend to, appreciate, and enhance the positive experiences in one’s life” (Bryant and Veroff, 2007, p. xi). When I first began working on the concept of savoring in 1980, psychologists universally recognized that when *bad* events occur, people do not automatically feel negative emotions—indeed, how much distress people experience in response to stressful events was presumed to depend on how people appraised and coped with these events. As the first-century, Greek philosopher Epictetus (c. 100 A.D./1983) observed, “What upsets people is not things themselves but their judgments about the things” (p. 13). Yet in 1980, the prevailing assumption in psychology was that when *good* events occur, people naturally feel positive emotions in response.

To me, however, what was true for bad events also seemed true for good events. As the French writer Francois de La Rochefoucauld (1694/1930) argued, “Happiness does not consist in things themselves but in the relish we have of them” (p. 51). In fact, I believed the two processes—coping and savoring—involved different sets of skills that were not mirror opposites:

Being able to handle adversity is vital in life, but having a capacity to cope seems not to be the same as having the capacity to enjoy life. In other words, just because people are not down, doesn’t mean they’re up. (Bryant and Veroff, 2007, p. 1).

I begin this article by reviewing the historical evolution of the concept of savoring, the development of measurement tools for assessing the conceptual components of savoring ability and savoring strategies, and the wide range of applications of savoring that have emerged in the psychosocial and health sciences. I then address important directions for future work on savoring, including promising new areas of application.

## Historical Evolution of the Concept and Measurement of Savoring

Before considering the future of work on savoring, it is useful to explain the origin of the construct and the evolution of its conceptual and operational definitions, and to provide a summary of current progress. As I describe below, the historical roots of savoring lie in large-scale survey research that I did in the early 1980s in collaboration with the late Joseph Veroff (23 November, 1929–30 September, 2007), during my post-doctoral fellowship at the University of Michigan. This research extended earlier work Joe and his colleagues had done on the dimensions underlying people’s self-evaluations of their own mental health (Veroff et al., 1962).

### Origins of the Concept of Savoring

#### The First Empirical Hints of Savoring

In our initial publication (Bryant and Veroff, 1982), Joe and I examined a diverse array of self-report measures of subjective adjustment that had been administered in two large, nationally representative, face-to-face, cross-sectional surveys of United States adults—one from 1957, the other from 1976—covering a broad spectrum of self-evaluations of “general happiness, worries, feelings of self-worth, symptoms of stress, recognition of problems experienced in work, marriage, and parenthood, and feelings of inadequacy and well-being attached to each of these roles” (Bryant and Veroff, 1982, p. 653). We then used exploratory and confirmatory factor analyses to test hypotheses about the structure of people’s self-evaluations of their subjective experience across the two surveys. Supporting *a priori* predictions, we found that three basic dimensions underlay appraisals of subjective experience: (a) positive affective evaluation, (b) negative affective evaluation, and (c) the evaluation of personal competence.

Because the evaluation of competence related more closely to negative affective evaluation than to positive affective evaluation, we concluded that this self-efficacy dimension reflected an evaluation of personal competence in dealing with *negative* experience more than an evaluation of personal competence in enacting *positive* experience. We noted other research (deCharms, 1968; Reich and Zautra, 1981) that suggested the perception of oneself as the locus of control for positive outcomes may be a critical, independent factor we had been unable to examine in our analyses, given that no measures of this dimension were available. We reported preliminary results that elaborated on this notion:

A factor analysis of the items added to the 1976 survey, in addition to the ones that were common to both years…distinguishes two types of personal competence—one centering on the capacity to cope with stress, the other on the capacity to derive positive experience. This distinction needs careful examination in future research and theory development (Bryant and Veroff, 1982, p. 672).

Highlighting the distinction between the capacity to handle negative experience and the capacity to derive positive experience laid the foundation for theory and research on savoring.

In a second article (Bryant and Veroff, 1984), Joe and I analyzed the wider range of measures collected in the 1976 national survey, seeking evidence for a four-factor model of people’s self-evaluations of subjective experience that involved evaluations of: (1) negative experience; (2) positive experience; (3) personal competence in handling negative experience; and (4) personal competence in deriving positive experience. Our findings supported several important conclusions. First, people’s self-assessments in response to *positively* focused items (e.g., happiness, value fulfillment, life satisfaction) differ from their responses to *negatively* focused items (e.g., anxiety, physical ill health, feelings of vulnerability). We termed the former items indices of psychological *well-being* and the latter items indices of psychological *distress*. Second, people make separate self-evaluations of sources of experience arising from the *external world* (e.g., feelings of vulnerability and uncertainty) and from *within the self* (e.g., lack of gratification and lack of self-confidence). These two conceptual distinctions—between (a) self-evaluations of *positive* versus *negative* experience and (b) self-evaluations of experiences originating in the *external world* versus originating in the *self*—were to become instrumental in guiding my initial work on savoring. In discussing the implications of our results, we emphasized that, “Future research that includes items directly focusing on perceived competence in handling difficulties and in deriving pleasant experiences is needed to determine whether these are, in fact, separate dimensions” (Bryant and Veroff, 1984, p. 124).

Just as coping embodies the cognitive and behavioral mechanisms through which people process *negative* events and regulate *negative* feelings in response to such events, we reasoned that there must be a parallel set of cognitive and behavioral mechanisms through which people process *positive* events and regulate *positive* feelings in response to positive events. At the time, however, there was no theory or evidence to support the existence of this hypothesized process, which we viewed as “the positive counterpart of coping” (Bryant and Veroff, 2007, p. 2).

#### In Search of the Missing Link

After co-authoring this second article, I accepted a position as Assistant Professor of Psychology at Loyola University Chicago in 1982 and embarked on an independent program of research, with the initial goal of testing the hypothesis that people’s self-evaluations of their ability to manage negative inner experience are distinct from their self-evaluations of their ability to manage positive inner experience. My first steps were to give the process of managing pleasant inner experiences a name, develop a measure to assess perceived competence in deriving positive feelings, and to test more directly the four-factor model of self-evaluations (i.e., negative external experience, positive external experience, competence in handling negative inner experience, and competence in deriving positive inner experience).

After considering a variety of possible terms for the process of managing positive inner experience (e.g., relishing, accentuating, capitalizing), I settled on the term *savoring*, because it “most vividly captures the active process of enjoyment, the ongoing interplay between person and environment” (Bryant and Veroff, 2007, p. 3), and it implies the act of mindfully appreciating something that is personally pleasurable. Laying the groundwork for a study to delineate savoring as a distinct phenomenon, I recast the original four-factor model of self-evaluations within the theoretical framework of perceived control (White, 1959; Rotter, 1966; Phares, 1976), by combining two prevailing conceptual distinctions: (1) people make separate self-assessments of their capacity to control *positive* versus *negative* outcomes (Gregory, 1978; Reich and Zautra, 1981); and (2) people’s controlling responses can be classified as attempts to change the *world*, i.e., primary control, versus attempts to change *oneself* to fit in with the world, i.e., secondary control (Rothbaum et al., 1982). As I noted in my article reporting this research:

By crossing primary-secondary control with positive-negative experience, a four-factor model of perceived control emerges that consists of self-evaluations of one’s ability to *(a)* avoid negative events (primary-negative control), *(b)* cope with negative events (secondary-negative control), *(c)* obtain positive events (primary-positive control), and *(d)* savor positive events (secondary-positive control). (Bryant, 1989, p. 774).

I then created a set of 15 items reflecting self-evaluations of the four types of perceived control and administered these items to a large sample of young adults along with the indices of subjective well-being and distress from Bryant and Veroff (1984).

Supporting hypotheses, confirmatory factor analyses revealed that responses to these items defined four underlying factors, two reflecting control over *external events* (Avoiding and Obtaining) and two reflecting control over *internal feelings* (Coping and Savoring). Further confirming hypotheses, Coping and Savoring were largely unrelated to each other (sharing only 7% of their variance), whereas Avoiding and Obtaining were more strongly interrelated (sharing 25% of their variance). In addition, Savoring, like Obtaining, related more strongly to indices of subjective well-being than to indices of subjective distress. Savoring, however, was significantly related to general happiness, whereas Obtaining was not. These latter results suggest perceived control over positive *emotions* has more to do with happiness than does perceived control over positive *events*. Confirming de La Rochefoucauld’s 1694 observation, clearly positive events alone are not enough to produce happiness. People also need to be able to attend to and relish (savor) the positive feelings that emerge from positive events. Follow-up work replicated these findings and extended their generalizability to adolescents (Meehan et al., 1993).

### The Development of Instruments for Assessing Savoring

#### The Savoring Beliefs Inventory

Encouraged by this evidence that people make global self-evaluations of their capacity to savor positive feelings, my next step was to refine the broad concept of perceived savoring capacity to include a finer-grained focus on temporal aspects of savoring positive experience. In particular, I hypothesized that people make separate but correlated self-assessments of their ability to savor (a) *future* positive events before they occur (anticipation), (b) *present* positive events while they are unfolding (savoring the moment), and (c) *past* positive events after they occur (reminiscence)—all three of which are essential components of the fundamental human capacity to attend to and appreciate positive experience.

In a series of six studies, I developed and presented evidence supporting the structural, discriminant, convergent, and predictive validity, as well as the internal consistency and temporal reliability, of the Savoring Beliefs Inventory (SBI; Bryant, 2003) as a self-report measure of people’s dispositional beliefs about their ability to appreciate positive experience in each of these three temporal domains. With this measure, respondents use a 7-point Likert-type scale (1 = *strongly disagree*, 7 = *strongly agree*) to rate their level of agreement with 12 positively worded and 12 negatively worded statements, in order to indicate how capable they believe they are of appreciating positive experiences through anticipating (8 items), savoring the moment (8 items), and reminiscing (8 items). The SBI provides not only separate subscale scores for Anticipating, Savoring the Moment, and Reminiscing, but also a global Total Score, as measures of people’s perceived capacity to appreciate positive experience.

Over the past two decades, the SBI has frequently been used in positive psychology. As cross-cultural research on savoring has grown, international researchers have translated and validated the SBI in a variety of languages, including *French* (Golay et al., 2018), *Spanish* (Robles et al., 2011), *Romanian* (Căzănescu et al., 2019), *Chinese* (Lin et al., 2011), *Japanese* (Kawakubo et al., 2019), *Korean* (Kim and Bryant, 2017), *Persian* (Aghaie et al., 2017), and *Turkish* (Metin-Orta, 2018). Bryant and Veroff (2007) also reported the development and validation of the Children’s Savoring Beliefs Inventory that is appropriate for respondents with at least a fifth-grade reading level.

A large body of research supports the reliability and validity of the SBI as a measure of savoring capacity (Smith and Bryant, 2017). In addition, systematic scrutiny of the individual SBI items strongly supports the instrument’s content validity (Kawakubo et al., 2019).

Beliefs about one’s ability to savor overlap conceptually with meta-cognitive beliefs about positive emotion. In particular, perceived competence in regulating positive emotions is correlated with meta-cognitive beliefs about the controllability and utility of positive emotions (Becerra et al., 2020). Indeed, believing that positive emotions are both controllable and useful may provide a cognitive foundation to support the acquisition of savoring skills.

#### Measures of Positive Emotion Regulation

Closely related to people’s beliefs about their ability to savor is the notion that while they are experiencing a positive event, individuals may engage in a variety of different thoughts and behaviors, which Bryant and Veroff (2007) termed *savoring responses* or *strategies*, that regulate their positive feelings.

To measure people’s use of specific cognitive and behavioral savoring strategies in response to positive events, Bryant and Veroff (2007) developed the Ways of Savoring Checklist (WOSC). Since then, a variety of other measures of positive emotion regulation have been created, including: (a) Feldman et al.’s (2008) *Responses to Positive Affect*, which measures emotion-focused and self-focused positive rumination and dampening thoughts in response to positive feelings; (b) Gentzler et al.’s (2010) self-report measure of emotion regulation strategies that maximize or minimize positive feelings in response to positive events; (c) Nelis et al.’s (2011) *Emotion Regulation Profile-Revised* (ERP-R), which assesses positive emotion regulation strategies in response to positive events; (d) Livingstone and Srivastava’s (2012) inventory of *Positive Up-Regulation Activities*, which assesses people’s use of three types of regulatory strategies (engagement, betterment, and indulgence) to create or maintain positive emotions; (e) Ramsey and Gentzler’s (2020) *Positive Events and Responses Survey for Adults*, which provides a global measure of savoring responses to six hypothetical positive events; (f) Weiss et al.’s (2015) *Difficulties in Emotion Regulation Scale–Positive*, which measures non-acceptance of positive emotions, and difficulties in goal-directed behavior and in behavioral self-control when experiencing positive emotions; (g) Wright and Armstrong’s (2016) *Inventory of Responses to Positive Affective States*, which provides subscales assessing characteristic responses to positive feelings; and (h) Preece et al.’s (2018) *Perth Emotion Regulation Competency Inventory*, which assesses the perceived ability to regulate positive and negative feelings.

It is beyond the scope of this paper to review all existing measures related to positive emotion regulation. Instead, I will briefly review two instruments that, in my opinion, provide the most comprehensive, multidimensional assessment of the degree to which people use specific thoughts and behaviors to amplify or dampen positive feelings in response to positive events—namely, the WOSC and the ERP-R. Other measures of positive emotion regulation certainly have their place in assessing global levels of amplifying and dampening (Gentzler et al., 2010), overall cognitive regulatory style (Feldman et al., 2008), emotion regulation ability (Preece et al., 2018), characteristic responses to positive affect (Livingstone and Srivastava, 2012), or problems in regulating positive feelings (Weiss et al., 2015). However, the WOSC and ERP-R are particularly useful in capturing both a broad- and narrow-band profile of the particular strategies people use to savor positive events.

#### The Ways of Savoring Checklist

The 60-item WOSC (Bryant and Veroff, 2007) assesses the degree to which people engage in *ten savoring strategies* (sharing with others, memory building, self-congratulation, sensory-perceptual sharpening, comparing, absorption, behavioral expression, temporal awareness, counting blessings, and kill-joy thinking) in relation to a recent positive event. Bryant and Veroff (2007) reported strong evidence supporting the reliability and discriminant validity of the WOSC as a measure of savoring strategies. The WOSC also includes an initial set of 12 questions assessing cognitive appraisals of the target positive event (e.g., its desirability, foreseeability, frequency of occurrence, and one’s degree of personal responsibility for the event’s occurrence) and 2 final questions assessing level and duration of enjoyment.

To create an abbreviated trait measure of savoring, Jose et al. (2012) used exploratory factor analysis to create a 30-item short form of the WOSC by selecting the three strongest loading items from each of the 10 savoring subscales, to produce global Amplifying and Dampening scales. To study savoring using experience sampling methodology, Jose et al. (2012) also modified the WOSC to create a three-item Momentary Savoring scale based on sharing with others, counting blessings, and sensory-perceptual sharpening—amplifying strategies consistently associated with stronger positive emotional reactions to positive events. Garland et al. (2019b) used four items from the WOSC, based on Jose et al.’s (2012) abridged measure, to form an adapted Momentary Savoring Scale for use as an indicator of a Positive Psychological Functioning latent variable, which mediated the impact of mindfulness training in reducing pain and risk of opioid misuse.

To promote cross-cultural research on the specific strategies people use to savor positive outcomes, researchers have adapted and validated versions of the WOSC in *Greek* (Pezirkianidis et al., 2021), *Hungarian* (Szondy et al., 2014), *Portuguese* (Carvalho, 2009), *Japanese* (Miyakawa et al., 2019), and *Korean* (Kim and Bryant, 2017). In addition, Hacin et al. (2014, July) created an abbreviated 20-item WOSC (with separate Amplifying and Dampening subscales) which they translated and validated in nine languages (Chinese, Czech, English, German, Hungarian, Portuguese, Russian, Slovene, and Spanish).

#### Emotion Regulation Profile-Revised

Paralleling work on the WOSC, Nelis et al. (2011) developed the 48-item Emotion Regulation Profile-Revised (ERP-R) as a self-report measure of savoring strategies. Using this instrument, Quoidbach et al. (2010a) distinguished among four types of *amplifying* strategies (behavioral display, being present, capitalizing, and positive mental time travel) and four types of *dampening* strategies (suppression, fault finding, distraction, and negative mental time travel). Whereas the ERP-R includes a wider range of *dampening* strategies than the WOSC, the latter includes a wider range of *amplifying* strategies than the former. Note that three of the ERP-R’s amplifying subscales and one of its dampening subscales are conceptually equivalent to counterparts of the WOSC—namely, behavioral display parallels the WOSC subscale of behavioral expression; capitalizing parallels the WOSC subscale of sharing with others (see Langston, 1994); being present parallels the WOSC subscale of absorption; and fault finding parallels the WOSC subscale of kill-joy thinking. Quoidbach et al. (2010a) presented evidence to support the validity of the eight ERP-R subscales, as well as global Amplifying and Dampening subscales, although they reported reliability data only for the two global subscales.

Despite the strong overlap in subscale content, the WOSC and ERP-R adopt quite different approaches to assess individual differences in the use of savoring strategies. Whereas the WOSC uses a continuous 7-point scale to measure the degree to which respondents engaged in individual thoughts or behaviors during a recent positive event, the vignette-based ERP-R uses a dichotomous (yes/no) response scale to assess whether or not respondents would engage in particular forms of amplifying or dampening in response to six hypothetical positive events.

Dispositional styles of savoring may sometimes reflect *positive rumination*, which Feldman et al. (2008) defined as “the tendency to respond to positive affective states with recurrent thoughts about positive self-qualities, positive affective experience, and one’s favorable life circumstances” (p. 509). People with bipolar disorder are particularly prone to engage in emotion-focused positive rumination (Johnson et al., 2008). Because positive rumination reflects what people “generally think and do” when they feel happy (Feldman et al., 2008, p. 511), positive rumination represents a more stable cognitive trait, whereas savoring strategies are cognitive and behavioral responses to specific positive events or emotions that vary more across situations (Bryant and Veroff, 2007).

### The Adaptive Utility of Savoring Strategies Is Context-Specific

It is important to note that particular strategies for emotion regulation are neither uniformly adaptive nor maladaptive—instead, whether or not a given strategy is adaptive may depend on the specific situation involved (Gentzler and Root, 2019). For example, after winning the first set of a five-set match in a tennis tournament, it might be *maladaptive* to celebrate, if it causes you to lose your competitive focus, or motivates your opponent to concentrate harder; and it might be *adaptive* to dampen your positive emotions in order to maintain your focus. In other situations, such as graduating from college, on the other hand, it might be *adaptive* to celebrate, if it solidifies personal meaning and builds a cherished memory; and it might be *maladaptive* to dampen positive emotions, if doing so makes one feel unfulfilled. Likewise, differences in cultural norms make Westerners more likely to display their positive emotions in public as a way to celebrate, whereas Eastern Asians are more likely to restrain their public displays of positive emotion to avoid making others envious (Choi et al., 2019). Thus, the adaptive value of savoring strategies is not universal, but rather is specific to particular situations and cultures (see Smith et al., 2019).

Consistent with this conclusion, research evidence indicates that greater regulatory diversity is associated with greater overall happiness among adults. In particular, the happiest individuals generally have a wider range of savoring strategies that they use across a greater variety of situations (Quoidbach et al., 2010a). Having a broader savoring repertoire and knowing when and how to use optimal combinations of various savoring strategies seems most beneficial.

### The Dramatic Growth in Research on Savoring

Empirical work on savoring has increased dramatically since Joe and I published our book, *Savoring: A New Model of Positive Experience* (Bryant and Veroff, 2007). One way to illustrate this expansive growth is to note representative examples of the wealth of savoring research that has flourished in the health and psychosocial sciences since the book appeared in print. Space limitations preclude an exhaustive review of current savoring-related applications. The examples listed below are merely the tip of the iceberg, and are intended to provide a general sense of the breadth of this work rather than a comprehensive survey of the literature.

#### Positive Psychology

Illustrating the many ways savoring has been applied, positive psychologists have developed an impressive array of cognitive-behavioral techniques for teaching clients how to savor as a way of reducing emotional deficits associated with depression (McMakin et al., 2011), hopelessness (Chen and Zhou, 2017), anxiety (Pereira et al., 2021), schizophrenia (Meyer et al., 2012), and anhedonia (Strauss, 2013). Researchers have also used savoring exercises to strengthen depressed people’s help-seeking intentions (Straszewski and Siegel, 2018), as well as to increase optimism (Biskas et al., 2018), happiness (Salces-Cubero et al., 2019), and life satisfaction (Smith and Bryant, 2019) among non-depressed individuals. Smith et al. (2014b) and Smith and Bryant (2017) review the large body of empirical evidence that illustrates savoring promotes positive psychological functioning.

#### Clinical and Health Psychology

Likewise, clinicians and health researchers have investigated the role of savoring in treating autism (Cai et al., 2018) and anxiety disorders (Eisner et al., 2009); preventing depression (Ford et al., 2016); reducing pain (D’Raven et al., 2015); helping people cope with stress (Samios et al., 2020), cancer (Hou et al., 2017), and acquired physical disability (Dunn and Brody, 2008); repairing the negative effects of state dysphoric rumination (Stone et al., 2020); reducing the link between marijuana use and marijuana problems (Luba et al., 2020) and reducing pain and opioid misuse risk (Garland, 2021). Research with older adults has also investigated the role of savoring in promoting resilience (Smith and Hollinger-Smith, 2015) and positive attitudes toward aging (Bryant et al., 2021), improving physical health (Geiger et al., 2017), buffering the deleterious effects of illness on subjective well-being (Smith and Bryant, 2016), and lowering cardiovascular reactivity and boosting agency (Borelli et al., 2020). Savoring has also been identified as a resource in bereavement (Permanadeli and Sundararajan, 2021), in lowering suicide risk (Klibert et al., 2019), and in protecting soldiers from the psychological effects of combat exposure (Sytine et al., 2018); and kill-joy thinking has been found to mediate the relationship between depression symptomatology and gambling disorder severity (Rogier et al., 2019). In addition, researchers have used savoring to increase people’s consumption of healthy foods (Coary and Poor, 2016), decrease overeating (Black and Areni, 2016), and promote healthy relationships with food (Batat et al., 2019).

#### Families and Close Relationships

Specialists in the area of children and families have examined the role of savoring in promoting healthy mother-child attachment (Gentzler et al., 2015), enhancing life quality for parents of young children (Burkhart et al., 2015), cultivating healthy family functioning (Cheung et al., 2019), helping children adjust to adolescence (Gentzler et al., 2013), helping caregivers adapt to stress in caring for chronically ill loved ones (Hou et al., 2016), and nurturing family ties for deployed military personnel (Borelli et al., 2014). Researchers have also studied the relationship between mothers’ levels of savoring and children’s adaptive skills (Song et al., 2019), the prospective influence of maternal modeling of savoring on child depression (Moran et al., 2019), and the detrimental impact of maternal depression on children’s savoring skills (Morrow et al., 2021). In addition, work on relational savoring has explored savoring as a predictor of relationship satisfaction (Lenger and Gordon, 2019), as an interpersonal resource for couples facing stress (Samios and Khatri, 2019), and as an intervention to enhance the quality of couples therapy (Antoine et al., 2020) and of long-distance romantic relationships (Borelli et al., 2015). In addition, Pitts (2019) has extended the concept of savoring into the fields of communication and language within the context of social relationships; and other work has studied the use of social network sites to boost savoring (Yu et al., 2020).

#### Organizational Behavior

Researchers in the field of organizational behavior have explored the role of savoring in reducing work-family conflict (Camgoz, 2014), boosting perceived job performance (Lin et al., 2011), enhancing organizational commitment among employees (Castanheira and Story, 2016), and improving workers’ mental and physical health (de Bloom et al., 2013). Other researchers have highlighted the benefits of savoring in the workplace for both businesses and their employees and have proposed strategies that organizations can use to enhance savoring among personnel (Fritz and Taylor, 2021).

#### Marketing

Marketing researchers have explored the role of savoring in maximizing the quality of guests’ experience in the hospitality industry (Chun, 2011), enhancing consumers’ enjoyment of recreational activities (Chun et al., 2017), understanding people’s decisions in planning retirement (Hardisty and Weber, 2020), and boosting the persuasiveness of advertising (Moore, 2010). Likewise, investigators in the field of leisure research have studied the role of savoring in enhancing tourists’ experiences (Yan and Halpenny, 2021) and emotional responses (Filep et al., 2013), in amplifying vacationers’ enjoyment of restaurants (Sthapit, 2017) and of Airbnb experiences (Sthapit et al., 2021), and in promoting therapeutic recreation (Carruthers and Hood, 2007) and meaningful engagement through leisure (Iwasaki et al., 2018). Savoring has also been used as a product design principle to trigger, amplify, and prolong user enjoyment (Pohlmeyer, 2014).

#### Education and Other Applications

Educational researchers have used savoring to foster student creativity (Lee et al., 2016), promote engagement and learning (Chang et al., 2021), reduce anxiety in foreign language classrooms (Jin et al., 2021), weaken the link between perfectionism and student distress (Klibert et al., 2014), and protect teachers from psychological burnout (Picado, 2012). Savoring has also been used as a resource in athletics (Doorley and Kashdan, 2021), and as a mechanism to understand how prayer enhances well-being (Crainshaw, 2014) and how time spent in nature boosts positive emotions (Sato et al., 2018).

#### Neuropsychology

There has also been an increased interest in the neuropsychology of savoring. Concerning savoring through reminiscence, for example, fMRI data reveal that enhanced activity in the striatum and medial prefrontal cortex is associated with increased positive emotion when recalling positive, relative to neutral, autobiographical memories (Speer et al., 2014). Concerning savoring the moment, EEG data indicate that participants instructed to savor monetary rewards show greater changes in reward positivity (i.e., a positive-amplitude deflection in event-related brain potential following reward feedback), compared to a control condition (Irvin et al., 2020). Along these lines, there is evidence that an intervention designed to enhance savoring increases neural responsivity to positive affect, which reduces opioid misuse (Garland et al., 2019a).

In the realm of clinical neuropsychology, there is also evidence that the depressive symptom of anhedonia (i.e., blunted response to pleasure and reward) reflects an underlying neurological deficit in the capacity to up-regulate positive emotion. In particular, when instructed to use cognitive appraisal to enhance emotional response to positive images, depressed individuals (compared to those who are not depressed) are unable to sustain activation of neural circuits in the nucleus accumbens (NAcc) and frontostriatal network that underlie positive affect and reward (Heller et al., 2009). These findings suggest that training depressed people to prolong engagement with tasks that activate the NAcc may be an effective behavioral treatment to enhance their ability to amplify positive emotion.

More recently, Wilson and MacNamara (2021) have provided the first empirical evidence that savoring is an effective and lasting means of increasing subjective and neural response to visual imagery. Participants instructed in how to savor positive pictures, compared to those who passively viewed these images, not only rated the pictures as more pleasant, but also showed increased picture-elicited late positive potential (LPP) in fronto-central and parieto-occipital regions of the brain (i.e., a neural marker of emotional arousal). Moreover, when viewing the same stimuli 20 min later (in the absence of instructions to savor), pictures that had been savored earlier were rated as more pleasant and more arousing and continued to elicit a larger LPP, compared to pictures that had not been savored.

### Evolution of the Conceptual Definition of Savoring

As with many constructs in psychology, the conceptual definition of savoring has evolved over time. Throughout this evolution, however, two bedrock concepts have remained central—namely, that savoring involves both (1) *some degree of mindful awareness of positive feelings*, as well as (2) *the management and regulation of positive experience*. I now briefly consider the evolution of theoretical perspectives on each of these key concepts.

#### Savoring as Meta-Awareness of Positive Feelings

An essential defining feature of savoring is that it “involves not just the awareness of pleasure, but also a conscious attention to the experience of pleasure” (Bryant and Veroff, 2007, p. 5). In other words, “savoring by virtue of its state of mindful meta-awareness is an experience of second-order consciousness” (Bryant and Veroff, 2007, p. 12). Reflecting this key conceptual element, my initial search for evidence of savoring as a human concern sprang from the notion that people are aware of their competence in deriving positive feelings and make self-evaluations of this ability that are distinct from positive feelings *per se* (Bryant, 1989). Along these lines, English essayist Samuel Johnson hinted at the vital nature of meta-awareness in savoring when he asserted in 1753, “Happiness is enjoyed only in proportion as it is known” (Hawkesworth et al., 1793, p. 216).

Likewise, the development of the Savoring Beliefs Inventory (SBI; Bryant, 2003) was also grounded in the notion that people attend to and are consciously aware of their capacity to derive positive experience. More specifically, the SBI is predicated on the assumption that people’s beliefs about their capacity to savor are reflections of their actual savoring ability. As Publilius Syrus (42 B.C./1856) observed over 2,000 years ago, “No man [*sic*] is happy who does not think himself so” (p. 53). Thus, savoring beliefs, by definition, involve a conscious awareness of one’s ability or inability to experience and manage positive experience.

Bryant and Veroff (2007) were the first to highlight explicitly a key conceptual component of savoring that must be always be present in order for savoring to exist—namely, a deliberate attentional focus on ongoing positive feelings. As Bryant and Veroff (2007) put it, “in savoring, people partially set a positive experience apart from their immediately attending self, such that the attending self interacts more directly with the focused experience” (p. 12). Thus, savoring must involve a mindful meta-awareness of positive experience (see Bryant and Smith, 2015), or else it is simply pleasurable enjoyment (Smith et al., 2014a).

The notion that people can experience pleasure without realizing it might at first blush seem logically impossible. Yet, evidence suggests people “can have experiences (experiential consciousness) without being contemporaneously aware of the nature of those experiences (meta-awareness)” (Schooler and Mauss, 2010, p. 244). For example, the brain may show valenced pleasure-displeasure responses to subliminal stimuli, to which people do not consciously experience an emotional reaction (Winkielman and Berridge, 2004). The willful regulation of emotion presupposes awareness of one’s emotional states (Price and Hooven, 2018).

#### Savoring as the Management of Positive Experience

Concerning savoring as the management of positive experience, I initially considered savoring to be a form of secondary-positive control over positive feelings that may stem from “beliefs about *(a)* cognitive or behavioral strategies that one can use to amplify or prolong enjoyment of positive events, *(b)* one’s ability to anticipate future positive outcomes in ways that promote a sense of pleasure in the present, *(c)* one’s ability to recall past positive events in ways that enhance present well-being, or *(d)* friends or relatives who can help one enjoy positive events, even if one cannot do so alone” (Bryant, 1989, pp. 775-776). Note that this initial formulation associates savoring with generating, intensifying, and prolonging positive feelings in response to positive outcomes (Kurtz, 2018), which implies that *savoring* (as emotional up-regulation) is conceptually the opposite of *dampening* (as emotional down-regulation)—a perspective that others have also adopted (e.g., Wood et al., 2003; Quoidbach et al., 2010a).

In my more extensive later work on savoring, in contrast, I “considered both amplifying and dampening responses to be efforts to regulate positive emotions that reflect different styles of savoring” (Bryant et al., 2011, p. 110). For example, people may dampen the joy of anticipation by downplaying upcoming positive events, in order to protect themselves from future disappointment (Norem and Cantor, 1986). Likewise, the cognitive savoring strategy of “kill-joy thinking,” which stifles positive feelings, is more common among East Asians than North Americans (Bryant and Veroff, 2007; Smith et al., 2019) and serves to regulate positive emotions in culturally normative ways. In a parallel fashion, although efforts to cope by “catastrophizing” one’s circumstances actually amplify distress, theorists nonetheless consider such reactions to be coping responses aimed at managing psychological distress (Keefe et al., 1989).

As illustrated above, the contemporary conceptualization of savoring has gradually evolved from a traditionally Western-cultural view of savoring as the capacity to create, intensify, and sustain positive experience, to a more nuanced cross-cultural view of savoring as the capacity to regulate positive emotion in ways that are personally and culturally appropriate, regardless of whether those regulatory efforts entail amplifying, dampening, or a combination of both. As Smith et al. (2019) observed, depending on individual, situational, and cultural factors, savoring may involve amplifying or dampening positive emotions, as well as increasing or decreasing their duration; and “greater enjoyment can occur through either an *increase* in savoring responses that amplify the duration and intensity of positive feelings or a *decrease* in responses that dampen positive feelings” (Smith et al., 2019, p. 151).

## Promising Directions for Future Theory and Research

Having reviewed the current state-of-the-art of work on savoring, I now focus on future directions for theory and research in this area. In particular, I highlight nine promising avenues for advancing theory, research, knowledge, and application in the years ahead: (1) exploring the dynamics of naturalistic savoring; (2) studying both reactive and proactive savoring; (3) integrating the perceptual judgments involved in the later stages of attending to and regulating positive experience (where past work has focused), as well as the initial stages of seeking out and noticing positive stimuli; (4) developing new ways to study people’s meta-awareness of positive feelings; (5) investigating the regulation of attentional focus in savoring; (6) understanding the developmental processes through which savoring skills and deficits evolve; (7) clarifying the role that savoring impairments play in the etiology and maintenance of psychopathology; (8) discovering new situational variables that enhance savoring; and (9) integrating the study of anticipation, savoring the moment, and reminiscence within individuals across time.

### Investigating Naturalistic Savoring

Because relatively little empirical work has focused on how savoring unfolds in everyday life, future work should systematically investigate the dynamics of naturalistic savoring. It is somewhat surprising that we currently know very little about when, how often, for how long, and in what ways people typically engage in episodes of savoring in their daily lives. In the earliest study of savoring in everyday life, Jose et al. (2012) assessed life events, savoring strategies, and mood at random times between 8 a.m. and 8 p.m. once daily for 30 days in a sample of undergraduates, and found that trait savoring produced higher levels of momentary savoring, which both mediated and moderated the impact of daily positive events on momentary happy mood; “habitual” savorers were more likely to maintain happy mood in the absence of positive life events, compared to people who did not consistently savor positive daily events.

Extending this naturalistic work, Heiy and Cheavens (2014) measured college students’ use of 20 positive and 20 negative emotion-regulatory strategies in response to positive or negative emotions experienced in the past 4 hrs, at random times during waking hours three times daily, and found that on average participants engaged in savoring strategies in response to about 7 positive emotion experiences across 10 days; they concluded that people may regulate positive emotion (compared to negative emotion) more often and with greater success. More recently, Colombo et al. (2021) measured undergraduates’ use of three categories of positive regulation—namely, “mindfulness and stimulus control for the category ‘attentional deployment’; broadening and counting blessings for the category ‘cognitive change’; and emotion expression and sharing for the category ‘response modulation savoring strategies’ ” (p. 5)—in reaction to positive events 3 times daily for 2 weeks, and found that the less individuals felt positive emotion at one point in time, the more they increased their use of amplifying strategies from this time point to the next. Thus, low momentary positive affect may motivate people to savor.

Clearly, more experience-sampling and daily-diary studies are needed to advance our understanding of how savoring occurs in everyday life. Rather than relying exclusively on retrospective assessments, however, this future work should study savoring as it occurs in real-time. As Bryant et al. (2011) noted, for example, prior cross-sectional and longitudinal research has ignored the temporal sequence in which multiple savoring responses occur: “Given a particular positive event, the same savoring responses arranged in different temporal orders might well produce different emotional consequences” (p. 116). For instance, if people use both amplifying and dampening strategies in response to the same positive event, we might expect to find recency effects, such that initial dampening followed by amplifying might produce greater positive affect, compared to initial amplifying followed by dampening (Bryant et al., 2011).

To advance our understanding of how savoring unfolds over time, I recommend future work focus on integrating the perceptual judgments that underlie the sequence of cognitive processes involved in searching for, noticing, attending to, and regulating positive experience (which comprises both positive stimuli and positive emotions). Prior research on savoring has focused almost exclusively on emotion regulation, and has largely ignored the initial stages of seeking out, detecting, and focusing attention on positive stimuli or feelings. Future work is also needed to disentangle how people regulate the *intensity* versus *duration* of positive emotions in everyday life (see Tugade and Fredrickson, 2007).

### Distinguishing Reactive and Proactive Savoring

Whereas most prior studies have investigated *reactive* savoring, which occurs spontaneously in response to positive events or feelings, future work is also needed on *proactive* savoring, which begins with the deliberate act of seeking out or creating positive experience, rather than passively waiting until one happens to notice positive stimuli. Along these lines, Quoidbach et al. (2015b) have extended Gross’s (1998) seminal process model of negative emotion regulation to the realm of positive emotion. Particularly relevant to proactive savoring are the up-regulation mechanisms of *situation selection* (in which people purposefully put themselves in situations likely to produce positive emotions) and *attentional deployment* (in which people choose to direct their perceptual attention to positive situational features).

There is reason to believe that proactive savoring is a valuable personal resource in managing one’s subjective well-being. For example, Catalino et al. (2014) found people who intentionally created conditions in daily life that were likely to produce positive emotions—such as gardening or spending time with a friend—reported greater happiness and life satisfaction and fewer symptoms of depression, compared to those who did not intentionally create such conditions. I propose that savoring mediates the benefits of situation selection. Given that enhancing awareness of positive experiences and strengthening positive emotion regulation can boost well-being (Smith et al., 2014b), a promising avenue for future research is to develop interventions to increase proactive savoring.

*Proactive* savoring requires the effortful allocation of time and energy to create a positive experience “from scratch” that one can savor, whereas *reactive* savoring, in contrast, merely requires awareness of an ongoing positive experience one has not intentionally created, but can savor. For this reason, proactive savoring may well be less common than reactive savoring across all three temporal domains. Proactive *savoring of the moment* fosters the joy of anticipation, whereas reactive momentary savoring fosters the joy of surprise. Proactive *reminiscence* serves to keep one’s storehouse of pleasant memories fresh and accessible, compared to only reminiscing reactively when external circumstances bring positive memories to mind. Likewise, proactive *anticipation* can heighten the joy of positive events both before and during their occurrence. Future work is needed to understand how to help people optimize their proactive efforts to set the stage for savoring. For example, prioritizing pro-hedonic goals may encourage optimal situation selection (Livingstone and Isaacowitz, 2015).

### Increasing Perceptual Sensitivity to Positive Stimuli

In relation to proactive savoring, people may be able to enhance their sensitivity to positive stimuli, in order to facilitate searching for and noticing things to savor. Neuroscience research shows that positive mood can broaden the scope of attention at both the perceptual and conceptual levels (Uddenberg and Shim, 2015). Indeed, people who are happy and satisfied with life preferentially attend to positive stimuli (Raila et al., 2015). Thus, savoring may in some ways be a self-sustaining process.

In the same vein, Jose et al. (2020) found that the use of amplifying strategies prospectively predicted an increase in the reported frequency of positive life events (i.e., an effect termed “uplift propagation”) over 3 months, whereas dampening responses did not significantly predict changes in uplift frequency over time. This uplift-propagation effect may result from a broadening and building of perceptual awareness of external positive events that savoring produces (Jose et al., 2020). Future experimental work should directly test the hypothesis that increasing how often people savor increases their reported frequency of positive events by making them more aware of existing uplifts, rather than by motivating them proactively to create more uplifts in their daily lives.

Given that humans possess finite attentional resources and that negative stimuli dominate our attentional field more than positive stimuli, people may not be naturally prone to savoring. Consistent with this conclusion, negative information is processed more quickly and thoroughly than positive, people exert more energy trying to eliminate bad moods than to induce good moods, and the effects of good events dissipate more rapidly than the effects of bad events (Baumeister et al., 2001).

The regular practice of savoring should strengthen the capacity to notice and attend to positive experience (Bryant and Smith, 2015). Indeed, neuroscience evidence indicates that “rewards ‘teach’ visual selective attention so that processing resources will be allocated to objects, features and locations which are likely to optimize the organism’s interaction with the surrounding environment and maximize positive outcome” (Chelazzi et al., 2013, p. 58). Future work should examine whether, “through the repeated practice of attending to positive stimuli and positive feelings, one may become habitually predisposed over time to seek out, attend to, and savor positive experience” (Bryant and Smith, 2015, p. 319).

People may also increase their attentional bandwidth in searching for “savorable” stimuli by avoiding things that would divide their attention. Future experimental work might investigate the effectiveness of cognitive and behavioral approaches, such as (a) blocking environmental distractions, (b) setting aside ego concerns, and (c) avoiding multi-tasking, time pressure, worrying, and complaining, as ways to increase signal-to-noise ratio and heighten sensitivity to internal and external positive stimuli. Specific types of techniques may work better to heighten sensitivity to different forms of positive stimuli—for example, *concentrating one’s gaze* to boost the impact of visual stimuli, or *closing one’s eyes* to intensify pleasing physical sensations, both being instances of sensory-perceptual sharpening (Bryant and Veroff, 2007).

### Measuring Meta-Awareness of Positive Feelings

An important future goal—and perhaps the most difficult—is to develop valid real-time methods of measuring the meta-awareness of positive feelings that is a necessary precondition for the process of savoring. Indeed, Bryant and Veroff (2007) argued that it is “necessary for one to be aware of one’s own enjoyment, or else there can be no savoring by our definition of the term” (p. 71).

As Smith and Bryant (2017) emphasized:

Research on savoring has sometimes relied on recalled enjoyment or time spent in pleasurable activity as a measure of active savoring, without directly assessing the meta-awareness of positive experience that lies at the heart of savoring. In the moment, however, people may be unaware of the enjoyment they later recall; or they may slow down simply to rest or relax, rather than to savor. Theorists and researchers should keep in mind that neither retrospective enjoyment nor momentary lingering directly captures the conscious awareness of ongoing positive feelings that is the quintessence of savoring. (p. 152).

Whereas researchers can readily measure behavioral and physiological components of savoring in a variety of ways, the meta-experiential component is far more challenging to assess. At present, the only well-established method we have to measure people’s internal experiences of awareness, cognition, and emotion is through retrospective self-reports obtained via open-ended qualitative questions or closed-ended rating scales or checklists. Yet, asking people to report their inner experience creates introspection and awareness that may change internal processes (Kassam and Mendes, 2013). Although researchers can ask participants to vocalize thoughts and feelings in real time, and use process-tracing methods such as verbal protocol analysis to study the content, order, intensity, and duration of these verbal expressions (Ericsson and Simon, 1993), such procedures are reactive and may well change the phenomena under study. Ultimately, EEG techniques may provide the definitive method of assessing meta-awareness.

### Regulating Attentional Focus While Savoring

In describing the focus of attention when one is savoring, Bryant and Veroff (2007) differentiated two general perceptual orientations in terms of whether the dominant attentional focus is outside (i.e., *world*-focused) or inside (i.e., *self*-focused) oneself. Both world- and self-focused attention can co-occur during the same savoring experience. As Bryant and Veroff (2007) noted: “Savoring processes involve noticing and attending to something positive, interpreting and responding cognitively or behaviorally to this stimulus (with savoring responses or strategies), experiencing positive emotional reactions as a consequence, attending to these positive feelings in an appreciative way, and often repeating this sequence of operations iteratively over time in a dynamic transactional cycle” (pp. 13-14).

Garland (2021) has adopted this idea in a “mindful savoring” exercise to help chronic drug users restructure reward processing from dependence on drug-related rewards to reliance on natural rewards, by teaching them to attend to the pleasing color, texture, scent, and feel of a rose, while also perceiving their reactions to the flower. When they become aware of good feelings, patients turn attention inward and focus on their internal experience and any affective associations that arise until these sensations fade, at which point they shift attention outward to appreciate the flower again. “Hypothetically, this toggling of exteroceptive and interoceptive attention on pleasant perceptions, sensations, cognitions, and emotions may overcome [habituation]…to intensify and prolong the pleasant experience” (Garland, 2021, p. 171).

On the other hand, there is also evidence that excessive self-monitoring can inhibit enjoyment. For example, people instructed to provide real-time evaluations of how happy they are while listening to music report less enjoyment than participants who simply listen to the music (Schooler et al., 2003). Thus, too much attention to good feelings, as opposed to just experiencing these feelings, may short-circuit savoring and disrupt enjoyment. As Ford (2019) observed, “paying close attention to one’s positive feelings may have an unfortunate side effect of attenuating those feelings” (p. 20).

The question naturally arises: At what point or in what contexts is savoring ineffective or counterproductive? Note that savoring requires one not only to *feel* good, but also to *reflect on* one’s good feelings. When reflecting on their good feelings, however, people may have insufficient attentional resources both to experience their affect and to evaluate it at the same time. As a result, anything that causes one to perseverate in making self-evaluations of positive feelings—e.g., inordinately valuing happiness (Mauss et al., 2011; Zerwas and Ford, 2021)—may make it harder for one to be aware of the affective experience itself. The more people wonder how happy they are or compare their feelings to what they wanted or expected to feel, the less happy they will be. As John Stuart Mill noted in 1873, “Ask yourself whether you are happy, and you cease to be so” (Mill, 1873, p. 100).

Regulating attentional focus while savoring is a complex mental task. Because the human capacity for conscious information processing is limited, external and internal attention are competing mental states—people can typically focus on either external or internal information alone one at a time and not simultaneously, although they can switch their attention quickly between external and internal foci (Benedek, 2018). Future research is needed to explore the naturally occurring dynamics of attentional focus during savoring experiences, as well as the determinants of shifts in outward- versus inward-directed attention.

Neuroimaging methods are likely to be crucial in this work, given substantial evidence that increased alpha activity, particularly in the right parietal cortex, is associated with internally directed attention (Cooper et al., 2003; Benedek et al., 2011). In addition, fMRI findings suggest that being strongly *externally* aware activates lateral fronto-parietal areas, whereas being strongly *internally* aware activates medial brain areas (Vanhaudenhuyse et al., 2011).

Future work might study structured savoring experiences to pinpoint reliable neuro-indicators of attentional shifts from positive stimuli (visual, auditory, olfactory, gustatory, haptic, or cognitive) to internal feelings. Researchers could randomly manipulate how long individuals are instructed to attend to: (a) positive stimuli before shifting attention to positive emotional responses, and (b) positive feelings before redirecting attention back to the initial positive stimulus. Other participants might simply be exposed to positive stimuli with no instructions about attentional focus, to assess naturally occurring experience as a standard of comparison.

Moreover, neuro-researchers could also test hypotheses about associations between neurological measures of attentional switching and hedonic LPP spikes. Including retrospective self-report measures in this work would enable hypothesis testing about links between different patterns of attentional switching and experienced positive affect in response to savoring.

### Understanding How Savoring Skills and Deficits Develop

Another major challenge for future work is to broaden our understanding of the processes through which children acquire skills or deficits in savoring (see Bryant et al., 2011). Traditional developmental models (e.g., Kopp, 1989; Calkins, 1994) presume the ability to regulate emotion is a process that begins during infancy and continues to develop through early childhood and adolescence, involving a combination of the *child’s* temperament and neuroregulatory systems, and the primary *caregiver’s* interactive style. Regarding the capacity for meta-awareness of emotion, psychoanalytic theory highlights the crucial development of “mentalized affectivity” in enabling individuals to identify, process, and express feelings, as well as to create new meaning by reflecting on affective experience (Jurist, 2005). Contemporary models of the acquisition of regulatory skills in early childhood also emphasize the pivotal role of parent-child attachment (Brumariu, 2015), parental socialization, observational learning, social referencing, and expressive language (Cole et al., 2010), as well as family emotion expressiveness and marital quality (Morris et al., 2007).

Supporting the importance of parental modeling in fostering positive emotion regulation, longitudinal research has prospectively linked *parents’* styles of savoring to savoring styles in both *children* (Moran et al., 2019) and *adolescents* (Fredrick et al., 2019). Related work has shown that levels of *parental* savoring in response to adolescents’ positive affect predict levels of *adolescent* savoring with respect to both amplifying (Nelis et al., 2019) and dampening (Raval et al., 2019). Other research with adults has linked avoidant-attachment to lower amplifying and greater dampening of positive affect in response to positive events (Goodall, 2015). Providing further insights, ecological momentary assessments reveal that when their children are present, mothers high in avoidance report lower positive emotion than mothers low in avoidance (Kerr et al., 2019). Extending this work, Palmer and Gentzler (2018) have provided experimental evidence that avoidant-attachment predicts lower savoring of interpersonal events, but is unrelated to savoring of non-interpersonal events.

Considered together, this body of evidence suggests that parents transmit styles of savoring to their children in the process of child rearing. More extensive longitudinal research is needed to identify the exact mechanisms through which modeling, parent-child attachment, and family dynamics shape specific savoring skills or deficits among children. To promote positive parenting, future work might develop interventions to raise parents’ awareness of the importance of savoring skills and teach them how to instill adaptive savoring in their children.

### Clarifying the Relationship Between Psychopathology and Impairments in Savoring

Dysregulation of positive emotions is important to understand because it not only deprives people of the benefits associated with positive emotions (du Pont et al., 2016), but also is related to various forms of psychopathology. Work on transdiagnostic clinical processes (e.g., Hechtman et al., 2013; Dalgleish et al., 2020) suggests that disruptions in positive emotion regulation generalize across a variety of diagnoses, although the specific ways these disturbances manifest themselves differ across disorders (Kring, 2008). For instance, problems in *up-regulating* positive emotions are a defining characteristic of depression (Carl et al., 2013); and difficulties in *down-regulating* positive feelings are a distinguishing feature of bipolar disorder (Gruber, 2011).

There appears to be a reciprocal relationship between savoring and psychopathology. On the one hand, savoring deficits, and the characteristic patterns of amplifying and dampening they involve, may influence the development of psychopathology. For instance, research indicates that stronger dampening of positive affect predicts increases in symptoms of depression (Raes et al., 2012; Raval et al., 2019); and interventions designed to help people up-regulate their positive emotions decrease depression (Taylor et al., 2017). Indeed, savoring-based approaches to treating depression may inform the development of transdiagnostic therapies (Silton et al., 2020).

On the other hand, psychopathology may also shape savoring. For example, the use of some savoring strategies may stem from *positive urgency*, a trait first identified by Cyders et al. (2007) that involves the dysfunctional tendency to engage in impulsive, risky behavior in response to positive emotions. Along these lines, positive urgency may increase the use of behavioral expression (i.e., expressing inner positive feelings through outward physical behaviors) as a generalized style of mood enhancement, which is predictive of problems with alcohol and gambling (Cyders et al., 2007).

Savoring capacities may also serve as a protective factor for people who have difficulties regulating negative emotion. Supporting this notion, there is evidence that the ability to savor buffers the relationship between poor coping skills and higher symptoms of anxiety (Chiu et al., 2020). For example, the ability to savor the moment may facilitate attentional disengagement from negative stimuli by increasing awareness of the positive aspects of an event, thereby augmenting the regulation of negative emotions (Chiu et al., 2020). Likewise, anticipating future positive events and savoring past positive memories may also strengthen efforts to cope, by engendering hope and optimism in the face of adversity and providing perspective and self-insight in relation to present problems.

Several formidable challenges await future work on savoring in relation to the etiology and maintenance of psychopathology. In particular, theorists and researchers face the daunting tasks of unraveling the complex processes through which (a) deficits in savoring contribute to the development and persistence of psychopathology and (b) psychological disorder impairs savoring. Applied clinical research is also needed to develop effective interventions to prevent or overcome savoring deficits, in order to enhance life quality for children, adolescents, and adults.

### Identifying New Situational Variables That Facilitate Savoring

We still have much to learn about how to increase people’s capacity to savor positive experiences. As Gregory et al. (2021) noted, “surprisingly little is known about what enhances savoring in the moment” (p. 1). Research suggests momentary savoring is heightened by mindfulness (Cheung and Ng, 2020), novelty (Mitas and Bastiaansen, 2018), awareness of temporal scarcity (Kurtz, 2008), and perceptions of uncertainty (Gregory et al., 2021), but diminished by impatience (House et al., 2014), perfectionism (Smith and Bryant, 2012), reminders of wealth (Quoidbach et al., 2010b), and a sense of experiential abundance (Quoidbach et al., 2015a).

Future theory and research are needed to expand our understanding of the conditions that increase or decrease not only savoring in the moment, but also savoring through anticipation and reminiscence. Although one might assume factors that make time appear more fleeting would enhance savoring by promoting a sense of temporal scarcity, such effects are not necessarily as straightforward as they seem. For example, mortality salience may either make one more attuned to emotionally pleasant stimuli (DeWall and Baumeister, 2007) or produce a numbing effect on emotional reactions to positive stimuli (Goode and Iwasa-Madge, 2019). Likewise, focusing on the imminent ending of a positive life experience may either increase enjoyment and appreciation of the time one has left (Kurtz, 2008) or produce greater sadness and worrying through anticipatory nostalgia (Batcho and Shikh, 2016). A vast and complex unknown awaits future investigation with respect to situational facilitators of savoring.

Further complicating matters, although novelty and uncertainty can enhance enjoyment, people tend to underestimate the pleasures of repeating positive experiences they have enjoyed before (O’Brien, 2019). In particular, we are prone to neglect pleasurable situational nuances that we can discover only through continued exposure. Indeed, people will pay costs to avoid repeating positive experiences in order to maximize their enjoyment, when in some contexts repetition would be equally or more enjoyable (O’Brien, 2019). I hypothesize that those who are more skilled at savoring are better able to seek out, find, and appreciate the unforeseen pleasures that await discovery in repeating familiar positive experiences.

### Integrating the Study of Anticipation, Savoring the Moment, and Reminiscence

Another challenging area for future research involves studying all three temporal forms of savoring simultaneously within individuals in everyday life. Evidence suggests the balance between episodic recall of positive experiences and positive anticipation promotes wisdom and a sense of purpose and direction in life (Webster, 2016). Very little work has tested integrative hypotheses about how the ways in which people look forward to upcoming positive life events influence their savoring and enjoyment of these events when they occur, and how the ways in which people anticipate and savor the moment shape their reminiscing about these events afterward. Yet, this “temporal cycle” of savoring is an ongoing process throughout our lives and sometimes occurs for more than one positive event at a time in the course of a single day.

Concerning the rich interplay between future-, present-, and past-focused savoring, anticipating an upcoming consumption experience can heighten enjoyment not only as it unfolds in real time, but also when it is recalled afterward (Chun et al., 2017). Likewise, the commonly used savoring strategy of memory building can both heighten enjoyment of a positive event in the present, as well as facilitate later reminiscence (Bryant and Veroff, 2007).

Adding further temporal complexity, “people can enhance current joy by looking forward to looking back on the present (*anticipated recall*) or looking back on having looked forward to the present (*recalled anticipation*)” (Bryant and Veroff, 2007, p. 174). Moreover, looking forward to future reminiscence may also intensify the joy of anticipation, just as looking back on past anticipation may intensify the joy of reminiscence. A great, untapped challenge is to learn how to help people harness this complex interplay of mental time-travel in savoring.

To reframe de La Rochefoucauld’s (1694) perceptive insight, the essence of happiness is not the good things in our lives, but rather the relish we get from savoring these good things. Perhaps the most important lesson that positive psychology can teach people is to become more aware of how to manage positive experience more effectively in their lives and to prioritize and set time aside for the practice of savoring. For as Robert Louis Stevenson (1881) aptly observed, “There is no duty we so much underrate as the duty to be happy” (p. 122). It is my fondest hope that theory and research on savoring will continue to flourish in the years ahead.

## Author Contributions

The author confirms being the sole contributor of this work and has approved it for publication.

## Conflict of Interest

The author declares that the research was conducted in the absence of any commercial or financial relationships that could be construed as a potential conflict of interest.

## Publisher’s Note

All claims expressed in this article are solely those of the authors and do not necessarily represent those of their affiliated organizations, or those of the publisher, the editors and the reviewers. Any product that may be evaluated in this article, or claim that may be made by its manufacturer, is not guaranteed or endorsed by the publisher.
